# The Multifaceted Therapeutic Mechanisms of Curcumin in Osteosarcoma: State-of-the-Art

**DOI:** 10.1155/2021/3006853

**Published:** 2021-10-11

**Authors:** Fatemeh Zahedipour, Monireh Bolourinezhad, Yong Teng, Amirhossein Sahebkar

**Affiliations:** ^1^Student Research Committee, Faculty of Medicine, Mashhad University of Medical Sciences, Mashhad, Iran; ^2^Department of Medical Biotechnology and Nanotechnology, Faculty of Medicine, Mashhad University of Medical Sciences, Mashhad, Iran; ^3^Department of Hematology and Medical Oncology, Winship Cancer Institute, Emory University School of Medicine, Atlanta, GA 30322, USA; ^4^Applied Biomedical Research Center, Mashhad University of Medical Sciences, Mashhad, Iran; ^5^Biotechnology Research Center, Pharmaceutical Technology Institute, Mashhad University of Medical Sciences, Mashhad, Iran; ^6^School of Pharmacy, Mashhad University of Medical Sciences, Mashhad, Iran

## Abstract

Osteosarcoma is a major form of malignant bone tumor that typically occurs in young adults and children. The combination of aggressive surgical strategies and chemotherapy has led to improvements in survival time, although individuals with recurrent or metastatic conditions still have an extremely poor prognosis. This disappointing situation strongly indicates that testing novel, targeted therapeutic agents is imperative to prevent the progression of osteosarcoma and enhance patient survival time. Curcumin, a naturally occurring phenolic compound found in *Curcuma longa*, has been shown to have a wide variety of anti-tumor, anti-oxidant, and anti-inflammatory activities in many types of cancers including osteosarcoma. Curcumin is a highly pleiotropic molecule that can modulate intracellular signaling pathways to regulate cell proliferation, inflammation, and apoptosis. These signaling pathways include RANK/RANKL, Notch, Wnt/*β*-catenin, apoptosis, autophagy, JAK/STAT, and HIF-1 pathways. Additionally, curcumin can regulate the expression of various types of microRNAs that are involved in osteosarcoma. Therefore, curcumin may be a potential candidate for the prevention and treatment of osteosarcoma. This comprehensive review not only covers the use of curcumin in the treatment of osteosarcoma and its anti-cancer molecular mechanisms but also reveals the novel delivery strategies and combination therapies with the aim to improve the therapeutic effect of curcumin.

## 1. Introduction

As cells inside the bone start to divide uncontrollably, bone tumors grow and create a mass of abnormal tissue. There are four major types of primary bone tumors including osteosarcoma, Ewing sarcoma family of tumors (ESFTs), multiple myeloma, and chondrosarcoma. Osteosarcoma is the second most common type of primary bone cancer and can be extremely aggressive. Several factors have been shown to contribute to the development of osteosarcoma, most commonly race, gender, and age. A higher incidence rate of the disease is registered among young males of African origin, which was supported by research conducted in Nigeria, Uganda, and Sudan [1]. Osteosarcoma most often occurs in developing bones in children and young adults. This tumor frequently results in lung metastases and is associated with respiratory system failure. Therefore, osteosarcoma shows an overall poor prognosis, since approximately 30% of patients develop lung metastasis. Established therapeutic strategies for osteosarcoma consist of radiation therapy, surgery, and adjuvant chemotherapy with several high-dose anti-tumor agents [2, 3].

Presurgical (neoadjuvant) chemotherapy accompanied by surgical resection of the primary tumor during the second or third cycle of the year-long chemotherapy regimen is the normal clinical procedure [4]. The most common agents used for the chemotherapy of osteosarcoma include doxorubicin, adriamycin, cisplatin, ifosfamide, methotrexate, and etoposide [5]. However, the five-year survival rate for patients with metastatic osteosarcoma is very low, at about 20% [6]. Moreover, chemotherapy can induce side effects and cytotoxicity and often promotes drug resistance. Also, patients who relapse after administration of currently approved chemotherapeutic drugs have few other options for chemotherapy and have dramatically decreased survival [7, 8]. Therefore, the development of novel treatment strategies is essential to improve the survival rate of patients as well as reduce toxicity and adverse effects.

Curcumin, extracted from *Curcuma longa* rhizomes (turmeric), exhibits various pharmacological activities including anti-inflammatory, anti-oxidant, and anti-cancer properties via modulating intracellular signaling pathways that control cell growth, inflammation, and apoptosis [6, 9–12]. The anti-cancer effects of curcumin are associated with its ability to induce cell apoptosis and suppress inflammation, metastasis, and angiogenesis as well as sensitize tumor cells to chemotherapy [13–15]. Anti-cancer effects of curcumin have been investigated in various types of cancers including breast, prostate, and colon cancer [16–22]. Another outstanding effect of curcumin is its ability to heal bone defects caused by tumor invasion or surgery [23, 24]. Taken altogether, curcumin appears to be an exceptional option for osteosarcoma therapy since it has a dual impact of suppressing osteosarcoma development while also repairing bone abnormalities. However, its therapeutic applications are limited at the present due to its weak solubility in water (11 ng/ml), rapid metabolism, and quick clearance from plasma [25]. As a result, synergistic combination methods as well as novel delivery systems have been used to increase the anti-cancer effects of this potent agent [26].

This review provides an updated summary of the potential effects of curcumin and its analogs in the treatment of osteosarcoma by modulating molecular signaling pathways and microRNAs expression. Furthermore, the current delivery curcumin systems to enhance its bioavailability, solubility, and therapeutic potential are reviewed and discussed.

## 2. The Effect of Curcumin on Different Signaling Pathways in Osteosarcoma

### 2.1. Curcumin and RANK/RANKL Pathway in Osteosarcoma

Osteoclasts are bone-specific macrophages that act as necessary mediators for bone degradation and osteosarcoma metastasis. Receptor activator of nuclear factor kappa-B ligand (RANKL), a tumor necrosis factor, is associated with the formation, activation, and function of osteoclasts as well as increased rates of metastasis and cell mobility in osteosarcoma. RANKL is produced by osteoblasts and attaches to the receptor activator of nuclear factor-*κ*B (RANK) that is expressed by giant osteoclasts. RANKL can act in an autocrine/paracrine/endocrine manner. The attachment of RANKL to its receptor by recruitment of TNFR-associated factors (TRAF; e.g., TRAF 1, 2, 3, 5, and 6) leads to the activation of Src/PLC*γ*, PI3K/Akt/mTOR, and MAPK (p38, JNK, ERK1/2) cascade that activates transcriptional activators including NF-*κ*B by TNF-induced I*κ*B kinase (IKK) and Fos/Jun and MITF and leads to the transcription of numerous effector genes. These effector genes include genes that participate in bone resorption such as cathepsin K, TRAP, and genes involved in cell adhesion and motility such as VCAM1, ICAM1, MMP9, and TRAF2, such that their activation leads to c-Jun N-terminal kinase activation and promotes osteoclastogenesis. Therefore, overexpression of RANK and RANKL is associated with an increased rate of metastasis and poorer outcomes for patients [27].

Curcumin has been shown to reduce skeletal tumor growth and lung metastases, which are often associated with an increased survival rate in osteosarcoma models. Curcumin is also able to increase cell mobility in osteosarcoma models. Osteotropic factors, such as glucocorticoids, 1, 25-(OH)2D3, IL-1, and PGE2 induce RANKL expression. For instance, the binding of IL-1 to the IL-1 receptor increases RANKL expression, stimulates the differentiation of osteoclast precursors into mature osteoclasts, and induces phosphorylation of cAMP response element-binding protein (CREB)/ATF2 by reactive oxygen species (ROS). Subsequently, it binds to the CRE domain of the RANKL promoter in primary bone marrow stem cells (BMSCs) and osteoblasts. This attachment increases the expression of RANKL to mediate osteoclast differentiation while inhibiting the differentiation of osteoblast [28].

Curcumin, as an anti-tumor agent, suppresses osteoclast formation in response to IL-1*α* by inhibiting the enzymes that generate ROS, thus inhibiting osteoclast formation [29]. Furthermore, curcumin-like denosumab, a monoclonal antibody for the treatment of osteoporosis, suppresses RANKL by inhibiting IKK and NF-KB activation or acts as a ROS scavenger to protect against bone destruction, decrease tumor burden, and also improve survival of osteosarcoma patients after chemotherapy [30].

By fostering macrophage polarization from the M1-type to the M2-type phenotype and attenuating activation of the Akt and NF-кB pathways, curcumin has a protective impact on RANKL-mediated osteoclastogenesis. Following the attachment of RANKL to its receptor, the classical osteoclastic pathways such as NF‐*κ*B, AKT, and MAPK are activated and lead to the activation of c-fos and NFATc1. NFATc1 increases the expression of osteoclastic-related genes as a master regulator of osteoclastogenesis and initiates the differentiation of osteoclast precursor cells. However, RANKL does not fully induce the differentiation of bone marrow macrophages without the activation of NFATc1. In turn, ectopic NFATc1 expression was found to control the differentiation of osteoclast precursor cells without stimulation by RANKL. Thus, preventing the release of proinflammatory cytokines and blocking the signaling pathways that are involved in osteoclastic signaling pathways may represent promising therapeutic targets for the treatment of osteosarcoma [31–34].

Yang et al. demonstrated that curcumin improves NF-*κ*B p65 phosphorylation, blocking the NF‐*κ*B pathway and Akt activation. Additionally, a remarkable decrease in the cellular levels of c‐fos and NFATc1 genes was observed following the treatment with curcumin. The authors also evaluated the immunomodulatory effect of curcumin because the state of macrophage polarization is important for the inflammatory microenvironment. M1‐type macrophages create a proinflammatory microenvironment. Osteoclastic signaling pathways are further activated by this proinflammatory microenvironment and facilitate the recruitment and differentiation of osteoclast precursor cells. Curcumin showed a protective impact on RANKL-mediated osteoclastogenesis by enhancing macrophage polarization from the M1-type to the M2-type phenotype and attenuating the activation of the Akt and NF-кb pathways. They further confirmed that curcumin ameliorated osteolysis and bone loss by utilizing an in vivo mouse calvarial destruction model [35].

**Figure 1:**
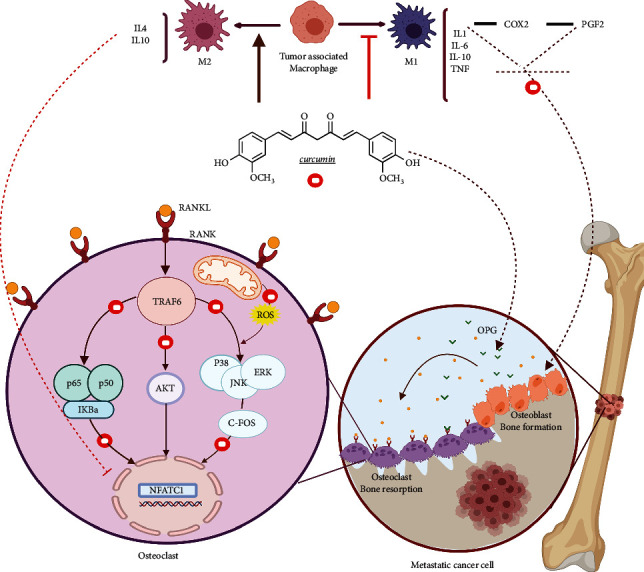
The role of curcumin in the regulation of the RANK/RANKL pathway in osteosarcoma.

Osteoprotegerin (OPG) is a glycoprotein member of the TNF receptor superfamily secreted by osteoblasts. OPG counterbalances the effect of RANKL by acting as a naturally occurring decoy receptor and inhibiting the interaction between RANK and RANKL, and thus, it can hamper the osteoclast formation and bone resorption. One of the most important clinical markers in patients with osteosarcoma is decreasing the levels of OPG and increasing the levels of RANKL protein expression. Upregulation of OPG suppresses the interaction of RANK-RANKL and osteolysis. Consequently, downregulation of RANKL/OPG may play an important role in the treatment of osteosarcoma. Curcumin has been shown to interact with RANK-RANKL interaction and lessen its ratio. Therefore, curcumin prevents the proliferation and invasion of osteosarcoma cells by decreasing RANK-RANKL-OPG levels [36] (Figure 1).

### 2.2. Curcumin and Notch Signaling in Osteosarcoma

The Notch signaling pathway plays essential roles in many types of cancers including osteosarcoma. Therefore, finding a potential agent that specifically targets Notch signaling could provide a promising treatment for osteosarcoma. Studies revealed that Notch pathway genes including Notch1, Notch2, Notch ligand DLL1, and Notch target genes including Hes-1, Hey-1, and Hey-2 were expressed in osteosarcoma cells. Moreover, the metastatic and invasive potential of osteosarcoma cells is associated with Hes-1 expression [37]. Notch1 has also been shown to interact with matrix metalloproteases (MMPs) that are critically involved in tumor cell invasion and metastasis processes [38].

Li et al. demonstrated that curcumin treatment resulted in a reduction of Notch-1, Hes-1, Hey-1, and Hey-2 mRNA levels in osteosarcoma cells. They concluded that curcumin regulates Notch-1 gene expression at both transcriptional and translational levels. They also found that expression of other Notch-1 downstream target genes including Hes-1 and cyclin D1 was suppressed following treatment with curcumin. In conclusion, their results demonstrated that curcumin exerts an inhibitory effect on osteosarcoma cell proliferation and invasion by inhibition of Notch-1 signaling, following the suppression of Hes-1, cyclin D1, MMP-2, and MMP-9 expression. These data suggest that downregulation of Notch-1 signaling by curcumin could be a potential treatment for osteosarcoma [39].

### 2.3. Curcumin and Wnt Signaling in Osteosarcoma

Overexpression of Wnt/*β*-catenin signaling plays a critical role in the development of osteosarcoma and can promote drug resistance [40]. In the canonical Wnt signaling pathway, the Wnt ligand binds to its receptor and Frizzled and low-density lipoprotein receptor-related protein 5/6 (LRP5/6), resulting in the release of *ß*-catenin from the APC/GSK-3*β* complex (axin-adenomatous polyposis coli/glycogen synthase kinase-3*β*). Subsequently, *ß*-catenin translocates to the nucleus and binds to Tcf/lef transcription factor, leading to activation of its downstream target oncogenes including c-Myc, cyclin D1, survivin, and MMPs that are contributing factors in tumor cell proliferation, metastasis, and apoptosis in many types of human cancers.

Overexpression of various Wnt components such as Frizzled receptor and Wnt ligands, secreted Frizzled-related protein 3 (SFRP-3), and Wnt inhibitory factor-1 (WIF-1) have been reported in osteosarcoma [41–43]. Moreover, an endogenous inhibitor of the Wnt pathway, Dickkopf-3 (Dkk3), affects intracellular *ß*-catenin resulting in suppression of metastasis and invasion in osteosarcoma cells [41]. Additionally, the expression of LRP-5, which is a coreceptor for Wnt, is associated with metastasis of osteosarcoma cells [41]. It has also been reported that dominant-negative LRP5 can inhibit the proliferation and metastasis of osteosarcoma cells via downregulating N-cadherin, MMPs, Twist, Slug, and Snail [44, 45]. Leow et al. evaluated the effect of PKF118-310 and curcumin on the Wnt/*β*-catenin signaling pathway. Their results revealed that curcumin targeted Wnt/*β*-catenin signaling and downregulated c-Myc, MMP-9, and cyclin D1. Therefore, curcumin could be a potential treatment for osteosarcoma that prevents proliferation, invasion, migration, and apoptosis in cancer cells [46].

### 2.4. Curcumin and Apoptosis Pathway in Osteosarcoma

Many studies have reported that curcumin is capable of inhibiting cell proliferation and inducing apoptosis by targeting several components within the cell. Induction of cell apoptosis and sensitization of drug-resistant tumor cells are very important for the treatment of cancers [47]. The ability of curcumin to cause apoptosis in cancer cells without cytotoxic effects on healthy cells helps explain the anti-cancer capacity of curcumin [48].

Boehning et al. demonstrated that the mRNA levels of inositol 1,4,5-triphosphate receptor type 1 (ITPR1) were increased in osteosarcoma cells following treatment with curcumin. The ITPR1 gene encodes the intracellular calcium release channel type 1 InsP3R that interacts with cytochrome c and mediates the calcium-dependent apoptosis pathway. Thus, curcumin can act as an anti-cancer agent in osteosarcoma cells by regulating ITPR1 and increasing apoptosis [49, 50].

Curcumin has been shown to arrest the cell cycle in G1/S and G2/M and activate caspase-3-mediated cell apoptosis. Important regulatory factors for the transition from G1/S and G2/M phase are cyclinD1 and cyclin B/cdc2. Treatment with low concentrations of curcumin resulted in the reduction of cyclin D1 and cell cycle arrest in the G1/S phase, while a high concentration of curcumin resulted in the reduction of cyclin B/cdc2 levels and cell cycle arrest in the G2/M phase [51].

Curcumin can also induce apoptosis by inducing caspase 3, a member of the cysteine-aspartic acid protease that is activated during apoptosis, irrespective of the specific death-initiating stimulus. Curcumin also inhibits the Bcl-2-mediated anti-apoptotic mitochondrial pathway by decreasing the cellular level of Bcl-2 and increasing BAX as key regulators of apoptotic cell death [52, 53].

Cleavage of poly-ADP-ribose polymerase 1 (PARP-1) as a substrate of caspase 3 and 7 is an important marker of cell apoptosis. In many cells, cleaved PARP can be used as a tool for the detection of apoptosis [54]. It has been shown that treatment with curcumin contributes to a substantial decrease in mitochondrial membrane potential and a rise in mitochondrial cytochrome C concentration [55]. Furthermore, curcumin treatment increased the expression of cleaved PARP and caspase 3, resulting in enhanced tumor cell apoptosis [53].

The 14-3-3 protein family acts as regulator molecules of protein-protein interactions, protein localization, and subcellular localization. 14-3-3*ε* is a member of this protein family that is involved in cell cycle regulation and apoptosis [56, 57] and is typically located on the nuclear matrix of osteosarcoma cells. Lu et al. found that treatment of human osteosarcoma MG-3 cells with curcumin-induced apoptosis via downregulation of 14-3-3*ε* in the nuclear matrix of apoptotic cells. They observed that 14-3-3*ε* was colocalized with p53, c-FOS, Bax, and Bcl-2 in the cytoplasm of osteosarcoma MG-63 cells. Following curcumin treatment, colocalization was decreased, leading to increased cell apoptosis [58].

**Figure 2:**
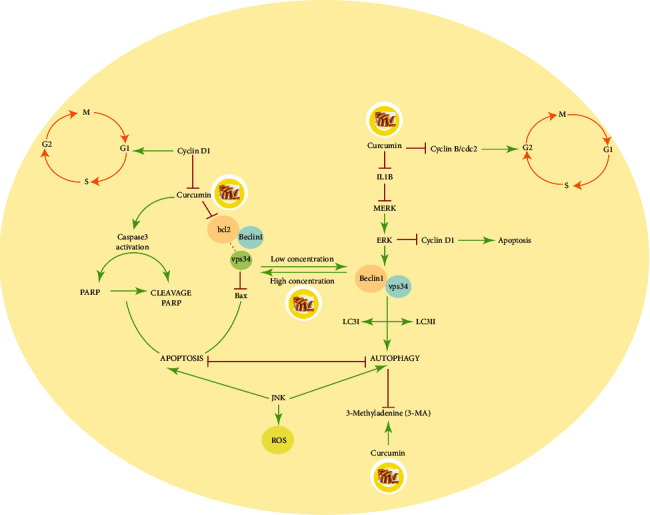
The effect of curcumin on apoptosis and autophagy signaling pathways in osteosarcoma.

Chang et al. conducted a study to evaluate the selective cytotoxicity of curcumin on osteosarcoma cells compared to normal osteoblasts. They demonstrated that curcumin showed higher selectivity to kill osteosarcoma cells in comparison with normal osteoblast cells. Their results demonstrated that curcumin can be a potential treatment for osteosarcoma with low cytotoxicity in healthy cells [59]. These results provide principal insights into the important role of curcumin in triggering apoptosis as an essential mechanism for the treatment of osteosarcoma (Figure 2).

### 2.5. Curcumin and Autophagy Pathway in Osteosarcoma

Autophagy is a pathway of cell degradation for the clearance of defective or unwanted proteins and organelles. To preserve cellular homeostasis and viability, the recycling of unwanted intracellular constituents often acts as an alternative energy source during times of metabolic stress. Autophagy promotes the extended survival of cancer cells with a defective apoptosis pathway. Defects in the autophagy pathway are also associated with increased tumorigenesis. In addition, autophagy can enhance both chemosensitivity and chemoresistance during the treatment of osteosarcoma under various conditions [47].

Curcumin can play an essential role in the autophagy pathway via the regulation of the c-Jun NH2-terminal kinase (JNK) pathway. JNK is a member of the mitogen-activated protein kinase superfamily and is a master protein kinase that plays an important role in osteoblast proliferation, differentiation, and apoptosis [60]. Curcumin-induced apoptosis can be enhanced in MG63 osteosarcoma cells by 3-MA, an autophagy inhibitor. 3-MA can reverse curcumin-induced upregulation of p-JNK and ATG5, which is an important gene in the autophagy process. Additionally, curcumin-induced p53 apoptosis can be upregulated by 3-MA. Therefore, curcumin can induce autophagy in osteosarcoma cells through the JNK pathway. Curcumin-induced autophagy also has an anti-apoptotic effect on osteosarcoma cells via regulation of Beclin1. Beclin1 is the interacting partner of Bcl-2 and forms a protein complex with phosphatidylinositol-3-kinase (PI3K) within the autophagosome. Beclin1 has been considered a crucial signaling regulator of autophagy. The expression of Beclin1 in osteosarcoma is significantly lower than in normal bone cells. Bcl-2, myeloid cell leukemia 1 (Mcl-1), and B-cell lymphoma-X large (Bcl-xL) can prevent autophagy by directly binding to the BH3 domain of Beclin 1/Atg6 under deficient Bcl2 through curcumin treatment. Therefore, Beclin1, which is normally dissociated from Bcl-2, is released to induce autophagy [61] (Figure 2).

### 2.6. Curcumin and JAK/STAT Signaling in Osteosarcoma

Aberrant activation of the JAK/STAT signaling pathway has been found to promote the development, proliferation, differentiation, migration, and survival of cancer cells in osteosarcoma [62]. JAK2 is involved in epithelial–mesenchymal transition (EMT) and mediates metastasis in cancer cells. Activated JAK2 can promote the phosphorylation of STAT3 and induces the expression of several genes involved in cellular proliferation. Curcumin can act as an anti-tumor agent by inhibiting the JAK/STAT pathway via suppressing JAK2 and STAT3 phosphorylation [63].

### 2.7. Curcumin and HIF-1 Signaling in Osteosarcoma

The high rate of metabolism in solid tumors leads to the expression of hypoxia-inducible factor-1*α* (HIF-1*α*) under hypoxic conditions. Overexpression of HIF-1*α* is a common occurrence in osteosarcoma and has been established as an independent prognostic biomarker [62]. Tumor progression is promoted under hypoxic conditions by activating signaling pathways involved in cell proliferation, angiogenesis, apoptosis, invasion, and metastasis. One of the downstream signaling components of HIF-1*α* is Notch-1 [64]. Aberrant expression of Notch-1 signaling has been reported in various types of cancers and has been correlated with cancer cell proliferation, survival, apoptosis, and differentiation [65].

Under hypoxic conditions, HIF-1 interacts with the intracellular domain of Notch1, which is generated by *γ*-secretase cleavage and translocate to the nucleus to activate Notch target genes. This cooperation is needed for tumor cells to maintain homeostasis in hypoxic conditions. Curcumin has been shown to inhibit hypoxia-induced HIF-1*α* expression by downregulation of Notch1 expression and acts as a potential anti-cancer agent for the treatment of osteosarcoma [32].

Zhang et al. revealed that tetrahydrocurcumin (THC), a main metabolite of curcumin, had a significant inhibitory effect on angiogenesis and HIF-1*α* expression in osteosarcoma cells. In addition, THC downregulates HIF-1 expression by suppressing Akt/mTOR and p38 MAPK pathways. These authors also observed that Akt/mTOR is an important upstream regulator of NF-*κ*B and GSK3*β*. Thus, NF-*κ*B and GSK3*β* might take part in the inhibitory effect of THC on HIF-1*α*. Moreover, they found that THC can trigger autophagy (by activating JNK and ERK), reverse epithelial to mesenchymal transition (EMT), and inhibit angiogenesis in a HIF-1*α*-related manner. However, the exact molecular mechanism underlying the THC-induced autophagy and mesenchymal to epithelial transition needs to be explored [66].

## 3. Curcumin and MicroRNAs in Osteosarcoma

MicroRNAs (miRNAs) are small noncoding RNAs comprised 20–24 nucleotides that have major roles in almost all biological pathways in various types of cells. MicroRNAs could act as either oncogenes or tumor-suppressor genes and could regulate the expression of various genes through multiple mechanisms. More than 2,000 microRNAs have been explored in humans. Alterations in microRNA expression profiles contribute to many cancers including osteosarcoma [69]. Curcumin has been found to act as a microRNA regulator and alters their expression in osteosarcoma. Yu et al. reported that curcumin treatment of MG-63 cells leads to upregulation of hsa-miR-138, has-miR-149, hsa-miR-181b, hsa-miR-193b, has-miR-339-5p, hsa-miR-671-5p, hsa-miR-22, and hsa-miR-124. It also results in the downregulation of hsa-miR-494, hsa-miR-186, hsa-miR-100, and hsa-miR-154. Additionally, they found that curcumin can suppress the proliferation and invasive ability of tumor cells by targeting Smad4, NF*κ*B p65, and cyclin D3 (regulatory factor of G1 to S phase of the cell cycle) genes through increasing the expression level of hsa-miR-138 [70].

Curcumin effectively decreases the expression of estrogen-related receptor alpha (ERR*α*) in osteosarcoma cells. ERR*α* overexpression decreases curcumin-activated apoptosis and scavenges the formation of ROS induced by curcumin, whereas ERR*α* silencing sensitizes osteosarcoma cells to curcumin and leads to enhanced inhibition of cell proliferation. Curcumin upregulates miR-125a and leads to suppression of ERR*α* gene expression. Curcumin-mediated tumor cell killing via activating the miR-125a/ERR*α* pathway induces enhanced levels of cleaved caspase 7, cleaved PARP, and apoptosis. In addition, downregulation of ERR*α* activates curcumin-mediated anti-tumor activity by ROS scavenging activity in osteosarcoma cells [47, 48].

miR-21 is a widely studied miRNA that is upregulated in several tumors and promotes tumor growth, resistance, and invasion [71]. miR-21 is mostly overexpressed in osteosarcoma, and the knockdown of miR-21 significantly reduces the invasion and migration of MG-63 cells. RECK (reversion-inducing cysteine-rich protein with kazal motifs) is a tumor suppressor gene, and a direct target of miR-21. RECK protein expression is negatively associated with miR-21 expression in human osteosarcoma tissues, indicating that miR-21 potentially controls RECK [72]. Zhou et al. revealed that curcumin can decrease the expression of miR-21 leading to upregulation of RECK expression. Cellular transfection of miR-21 mimics showed decreased levels of RECK. This finding indicates that curcumin induces apoptosis via the miR-21/RECK axis. In addition, curcumin deregulated miR-21 and upregulated RECK to inhibit Wnt/*β*-catenin signaling pathways and lead to inhibition of osteosarcoma cell proliferation. The Wnt signaling pathway is associated with various biological functions and is involved in tumor growth and chemoresistance [73].

Sirtuin 6 (SIRT6), which is a member of the sirtuin family of NAD^+^-dependent enzymes, plays many important roles and has multiple enzymatic activities including monoadenosine diphosphate (ADP) ribosylation, deacetylation, and defattyacylation [74]. Investigations revealed that SIRT6 acts as a tumor suppressor or oncogene in many types of human cancers [75]. SIRT6 is also upregulated in osteosarcoma cells. MMP9 levels are regulated by SIRT6 probably through the MEK-ERK1/2 pathway and lead to enhanced cell migration and invasion. Therefore, SIRT6 may function as a prognostic factor and a drug target for osteosarcoma patients [76]. It has been demonstrated that curcumin can inhibit SIRT6 expression through upregulating the expression of miR-33b-5p expression resulting in the inhibition of osteosarcoma cell migration and invasion. As a result, curcumin may be a potential therapeutic agent for the management of osteosarcoma [77, 78].

## 4. Curcumin Analogs as Synthetic Derivatives of Curcumin in Osteosarcoma

Considering the approved safety profile and a broad spectrum of anti-cancer effects of curcumin, this natural compound could be a potential therapeutic agent for osteosarcoma. Therefore, in recent years many curcumin analogs and derivative compounds have been investigated. To improve the bioavailability of curcumin in circulation, researchers have synthesized curcumin analogs. (Z)-3-hydroxy-1-(2-hydroxyphenyl)-3-phenylprop-2-en-1-one (DK1) is a lower molecular weight analog of curcumin that was confirmed to have cytotoxic effect on breast cancer cells via inducing cell cycle arrest and apoptosis. Furthermore, DK1 downregulates the mRNA expression of Cdk 2 and cyclin A (regulators of S phase cell cycle progression) and induces cell cycle arrest at S phase as well as curcumin, as indicated previously [79]. The effect of DK-1 on osteosarcoma has also been studied. The results showed that DK1 successfully inhibited the proliferation of osteosarcoma cell lines in a dose-dependent manner. Moreover, DK1 displays better cytotoxic effects toward osteosarcoma cell lines in comparison with natural curcumin. Additionally, DK1 activated apoptosis in an intrinsic manner (mitochondrial-dependent) by inducing proapoptotic proteins such as procaspase 3, cleaved caspase 3, Bax, cytochrome c, Fas, HTRA2/Omi, and SMAC/Diablo. It also inhibited anti-apoptotic proteins such as HO-1/HMOX1/HSP32 in osteosarcoma cells [80].

Another curcumin analog, CH-5 has the *α*,*ß*-unsaturated monoketone replaced with an *α*,*ß*-unsaturated diketone and has been shown to exert better anti-cancer and pharmacokinetic profile compared to curcumin. CH-5 was observed to reduce cell viability through the induction of apoptosis, migration, and invasion in the human gastric cancer cell line [81]. Its potential anti-tumor effect against osteosarcoma has been also investigated by Lima et al. They reported that CH-5 exerts its anti-tumor activity by stabilization of p53 and downregulation of sp1 protein leading to activation of apoptosis via modulation of DNA methyltransferase 1 (DNMT1) and damage-inducible 45 alpha gene (Gadd45a) expression. They also found that CH-5 inhibits osteosarcoma cell migration and invasion by decreasing MMP-2 and MMP-9 protein levels [82, 83].

FLLL32 is a diketone analog of curcumin that decreases DNA-binding activity and expression of STAT3 and induces apoptosis in osteosarcoma cell lines. FLLL32 is produced by the replacement of the two hydrogen atoms on the central carbon of curcumin with a spiro-cyclohexyl ring. It was suggested that this alteration would confer greater stability and specificity for STAT3 than curcumin by improving the interaction of FLLL32 with the Src homology-2 (SH2) domain of STAT3. Treatment of osteosarcoma cells with FLLL32 decreased the expression of survivin, VEGF, and MMP2 at both mRNA and protein levels with a concomitant decrease in STAT3 levels. This reduction of total STAT3 resulted, in part, from the ubiquitin-proteasome pathway [84].

It has been shown that the viability of human osteosarcoma HOS and U2OS cancer cells was reduced by curcumin, dimethoxy curcumin (DMC), and bisdemethoxycurcumin (BDMC). In addition, curcumin, DMC, and BDMC mediated HOS cell apoptosis via Smad 2/3 induction or Akt signaling pathway inhibition. Also, the combination of curcumin, DMC, and BDMC synergistically decreased the viability of cells and the development of colonies and increased apoptosis in HOS cells compared to either two or a single agent. It has been reported that to differentially enhance anti-metastasis function, the exclusion of one or two methoxy groups in the ortho position on the aromatic curcuminoid ring is essential, with BDMC and DMC being more potent than curcumin. Curcumin, dimethoxycurcumin, and bisdemethoxycurcumin prevent cancer cell invasion by downregulation of urokinase plasminogen activator (uPA) and matrix metalloproteinases (MMPs). The regulation of uPA and MMPs has an essential impact on cancer cell invasion via cleavage of the extracellular matrix [85].

Surprisingly, Huang et al. observed that curcumin and DMC induced the apoptosis of osteosarcoma cells by activating the Smad2/3 signaling pathway, while BDMC did not. Importantly, caspase 3 activation and p-Smad2/3 phosphorylation in osteosarcoma cells were significantly induced by curcumin and DMC resulting in apoptosis of caspase-mediated osteosarcoma cells. In addition, activation of Smad2/3 and caspase 3/9 led to overexpression of inhibitor of growth family member 5 (ING5), a tumor suppressor gene, and induction of apoptosis [86].

L48H37 is a new curcumin analog that can inhibit human osteosarcoma cell migration and invasion via inhibition of uPA or uPA receptor (uPAR) expression. UPA or uPAR overexpression is a malignancy characteristic and is associated with tumor progression and metastasis. L48H37 can reduce the phosphorylation of STAT3, JAK1, JAK2, and JAK3 in osteosarcoma cells without any effect on the phosphorylation of p38, ERK, and JNK. To sum up, L48H37 inhibits the invasion and migration of osteosarcoma cells through suppression of uPA expression and the JAK/STAT signaling pathway [87].

## 5. Curcumin Delivery Systems to Osteosarcoma Cells

Several approaches have been pursued to overcome barriers to using curcumin since the solubility of curcumin in water is very low and the rate of degradation in an alkaline state is very high. The use of delivery systems such as hydrogels, liposomes, dendrimers, and micro-/nanoparticles to encapsulate the hydrophobic curcumin in its hydrophobic core has been investigated [88]. The amphiphilic peptide C18GR7RGDS is a nanoparticle carrier of curcumin in an aqueous solution with self-assembly characteristics in water and phosphate-buffered saline that shows a selective cytotoxicity effect against osteosarcoma cells compared to normal cells. Thus, curcumin-loaded spherical amphiphilic nanoparticles (APNPs) may be a promising choice for the treatment of osteosarcoma. However, further investigation is needed to confirm the stability of APNPs under various pH conditions, especially alkaline pH [89].

Natural delivery vehicles have been shown to have greater biocompatibility and less immunogenicity. Casein, a protein found in milk, has been used as a natural carrier of bioactive agents. Casein was used as a reducing and capping agent for the biogenic synthesis of zinc oxide nanoparticles (ZnONPCS). This nanodrug delivery system has bactericidal and anti-cancer activity through the generation of intracellular ROS in breast cancer, cervical cancer, osteosarcoma, and myeloma cell lines [90]. The combination of this nanoparticle and curcumin achieved high treatment efficiency in cancer cells. ZnONPCS-curcumin treatment showed greater cytotoxic and anti-proliferative activity in cancer cells compared to cells treated with ZnONPCS and curcumin alone. Furthermore, the dispersibility and bioactivity of curcumin were increased by this conjugation. These findings suggest ZnONPCS-curcumin as a promising therapeutic agent [91, 92].

While curcumin is highly cytotoxic against cancer cells, its effective functional application is restricted by its hydrophobicity and rapid degradation at physiological pH. Encapsulation of curcumin with starch micro-/nanoparticles can improve release by changing the curcumin structure from crystalline to amorphous or disordered crystalline structure. Curcumin encapsulation improves its cytotoxic effect on MG-63 osteosarcoma cells because of its sustained release. However, it has no cytotoxic effect on human adipose mesenchymal stem cells [93].

Another formulation of curcumin for improved bioavailability is PLGA nanoparticles loaded with curcumin (Cur-NPs), which have been tested on osteosarcoma cell lines. Cur-NPs showed a selective cytotoxicity effect on abnormal cells while having a less cytotoxic effect on normal cells. Cur-NPs also induce the intrinsic apoptosis pathway through Akt-Bad signaling by activation and expression of caspases-3, caspases-7, and caspases-9, cytochrome c, Apaf-1, Bad, and suppression of the protein expression level of p-Akt [94].

To improve the bioavailability of curcumin, different lipid modifications have been developed in its formulation that would enhance its therapeutic potential. To determine curcumin tolerability and dose-plasma concentration relationship in a clinical study of late-stage osteosarcoma patients, Gota et al. tested solid lipid curcumin particle (SLCP) formulation versus unformulated curcumin in healthy volunteers. Their results showed that the relative bioavailability of SLCP was significantly enhanced in comparison with generic curcumin extract. Moreover, their results indicated the potential for sustained release of the drug when using SLCP as a lipid-based formulation [95].

Phospholipid vesicles consisting of one or more concentric lipid bilayers enclosing separate aqueous spaces are described as liposomes. Recently liposomes have been used as delivery systems for poorly water-soluble drugs such as curcumin to enhance their bioavailability and therapeutic efficacy. Withers et al. have evaluated the effect of Lipocurc™ (liposome-encapsulated curcumin) relative to free curcumin on the viability of mammary carcinoma, melanoma, and canine osteosarcoma cell lines. Lipocurc™'s ability to inhibit endothelial cell viability, migration, and tube formation has also been determined in vitro and in vivo. Their results indicated that Lipocurc™ inhibited proliferation, migration, and angiogenesis by inhibiting endothelial cell viability of canine osteosarcoma cell lines. The anti-cancer effects of Lipocurc™ were at least equivalent to that of curcumin on canine cancer cell lines [96].

The 2-hydroxypropyl-*γ*-cyclodextrin/curcumin-liposome complex has been also used to enhance the solubility of curcumin. Liposomal curcumin initiated caspase-dependent apoptotic cell death in vitro, whereas DMSO-curcumin induced autophagic cell death. Additionally, DMSO-curcumin-loaded induced an immune cell response following the initiation of autophagy, whereas using liposome and cyclodextrin for encapsulation of curcumin did not trigger an immune response and induced cell apoptosis. Therefore, curcumin-encapsulated liposome and cyclodextrin could be potential anti-cancer delivery vehicles for the treatment of various cancers including osteosarcoma [97].

Among polymeric delivery systems, one of the most popular polymers is polyethylene glycol (PEG) due to its ability to form copolymer structures with other types of polymers as well as other special characteristics such as hydrophilicity, transparency, deformability, nontoxicity, nonimmunogenicity, and biocompatibility. Curcumin encapsulated in natural biodegradable polyphenol hydrophilic copolymer y (polyethylene glycol)-poly(D,L-lactide-co-glycolide)/poly(*ε*-caprolactone) (mPEGPLGA/PCL) and (polyvinyl alcohol-polyethylene glycol − PVA-PEG) showed a synergic cytotoxic and anti-proliferation effect in osteosarcoma cells by decreasing the expression of CMYC, as a major protooncogene. Furthermore, this formulation can decrease the expression levels of MMP7, which correlates with metastatic features in cancers, thus preventing the invasion of osteosarcoma cells [88, 97].

Three-dimensional printed (3DP) tissue engineering scaffolds are the best choice for the substitution of bone grafts due to their control over geometry, connectivity, and chemistry of pores. The incorporation of anti-cancer drugs and growth factors can enhance the therapeutic efficacy of these scaffolds. In this regard, Sarkar et al. synthesized 3DP from tricalcium phosphate (TCP) ceramics. They encapsulated the hydrophobic curcumin into a liposome and incorporated the liposome within a 3DP-TCP scaffold. The scaffold can act as a mechanical support for the attachment of cells. In addition, persistent drug release from the liposome helps achieve enhanced bioavailability, improved therapeutic index, and decreased toxicity. Their results revealed that a 3DP bone tissue-engineered scaffold containing curcumin-encapsulated liposomes suppressed osteosarcoma cells by 96% in comparison with untreated samples in vitro. It also promoted proliferation, adhesion, and formation of filopodial prostheses on the scaffold surface and showed no cytotoxicity toward healthy osteoblast cells [63]. In another study, these researchers utilized poly(*ε*-caprolactone) (PCL), polyethylene glycol (PEG), and polylactide coglycolide (PLGA) as polymeric systems to enable the persistent release of curcumin from the hydroxyapatite matrix as well as to increase its bioavailability. Furthermore, they synthesized 3D printed interconnected macroporous *ß*-TCP scaffolds loaded with curcumin-PCL-PEG to evaluate the effects of curcumin on bone regeneration in vivo. Their results indicated that curcumin-loaded TCP led to enhanced bone regeneration after 6 weeks. Moreover, the complete formation of mineralized bone enhanced from 29.6% to 44.9% in curcumin-coated scaffolds in comparison with pure TCP [98, 99].

Among polymeric micelles, surfactant micelle solutions are good choices for curcumin delivery, since they show high selectivity and lack toxicity to normal cells. Hydrophobic polysaccharides routinely have been used for the encapsulation of curcumin. Hyaluronic acid-octadecanoic acid (HA-C18) are amphiphilic copolymer micelles that are able to assemble by themselves into micelles. They have been used to encapsulate curcumin. The in vivo delivery of curcumin-loaded HA-C18 demonstrated anti-tumor activity with sustained release of curcumin and high curcumin loading to osteosarcoma tissue. Therefore, this curcumin delivery strategy may be a potential therapy for osteosarcoma [100].

Recently, the use of implantable biomaterials loaded with drugs has appeared promising due to both their cancer cell‐killing ability and excellent bioactivity. Zhang et al. produced a functionalized titanium-based implant biomaterial manufactured by loading curcumin into a cyclodextrin-based polymer (pCD) modified titanium dioxide (TiO2) nanorod arrays. In addition, a polydopamine (pDA) supported film was used to ensure the tight attachment of the pCD as the first coating layer on the surface of the TiO_2_ nanoarrays. The pCD coating function as a reservoir for curcumin led to efficient loading of drug and continuous release of anti-cancer agent. According to the results, this curcumin-modified surface significantly triggered ROS formation resulting in mitochondrial dysfunction and induction of apoptosis in osteosarcoma cells in vitro and in vivo [101].

Altogether, encapsulating curcumin in these different carriers may result in a considerable increase in its anti-cancer effects. The biocompatibility and anti-cancer effectiveness of the aforementioned nanocarriers have been partially confirmed in several settings. However, more in vivo and clinical investigations are required to allow them to deliver to cancer patients safely.

## 6. Curcumin-Based Combination Therapy in Osteosarcoma

Over the past decades, combination therapies have been used for high-grade osteosarcoma [102]. Osteosarcoma, like other solid tumors, comprises a highly heterogeneous cell population that differs in many aspects such as karyotyping, proliferation rate, antigenicity, and chemoresistance. Thus, treatment of high-grade osteosarcoma requires less toxic combination therapy. Several combination therapies have been identified for the treatment of osteosarcoma, including bisphosphonates with paclitaxel/doxorubicin/gemcitabine and ifosfamide + methotrexate + cisplatin + doxorubicin [103, 104]. Recently, many studies have been conducted to evaluate the synergistic cytotoxic effects of curcumin in combination with other therapeutic agents.

Dhule et al. studied the anti-tumor potential of curcumin and C6 ceramide in osteosarcoma cells. They evaluated three liposomal formulations including C6 liposomes, curcumin liposomes, and C6-curcumin liposomes. They found that osteosarcoma cells that were treated with C6-curcumin liposomes showed 1.5 times enhanced cytotoxic effects compared to curcumin liposomes alone. Moreover, they reported that liposomes containing only curcumin-induced G2/M cell cycle arrest via overexpression of cyclin B1, whereas liposomes containing only C6 induced G1 cell cycle arrest via downregulation of cyclin D1. Moreover, liposomes containing both C6 and curcumin induced G2/M cell cycle arrest and displayed a combined effect on the expression levels of cyclin D1 and cyclin B1. To increase the half-life of liposomes in the plasma and targeted delivery of liposomes in vivo, they used PEG and folate, respectively. The C6-curcumin pegylated liposomes containing folate resulted in a significant reduction in tumor size [105].

Pancratistatin (PST) is a natural compound with anti-neoplastic activity by inducing apoptosis in cancer cells in a mitochondrial manner. This natural compound shows high selectivity for cancer cells, while normal cells are less sensitive to it [106]. Due to the low quantity of PST in natural sources, several PST analogs have been synthesized such as JC-TH-acetate-4 (JCTH-4). They induce ROS formation, inactivate MMPs, and induce mitochondrial release of apoptosis-inducing factor (AIF) and endonuclease G (EndoG). Furthermore, JCTH-4 induces autophagy in osteosarcoma cells. A combination of curcumin and JCTH-4 has been found to exhibit more selective cytotoxic effects in comparison to JCTH-4 and curcumin treatment alone [107].

Resveratrol (trans-3,5,4′-trihydroxystilbene) is a polyphenolic phytochemical with anti-oxidant, anti-inflammatory, anti-cancer, anti-aging, and anti-tumor activities derived from many natural sources such as grapes, berries, plums, peanuts, and pines [108]. Diallyl disulfide (DADS) is also an anti-cancer organosulfur compound derived from garlic (*Allium sativum*) with some known effects including anti-proliferation, anti-angiogenesis, histone modification, anti-oxidant, regulation of cell cycle arrest, and induction of apoptosis [109]. It has been demonstrated that resveratrol and DADS are capable of inhibiting the growth of osteosarcoma cells when administered as single agents [110, 111]. Masuelli et al. reported that combinations of curcumin with resveratrol or DADS can be more effective in inducing apoptosis than treatment with curcumin as a single therapeutic agent. They also mentioned that curcumin treatment of osteosarcoma alone or in combination with other drugs is independent of p53 activity, suggesting the potential of curcumin in the treatment of p53 deficient cancers [112].

Amino naphthoquinones are polycyclic aromatic hydrocarbons with an anti-cancer activity that act as anti-proliferative components via various mechanisms such as ROS generation, regulation of p53, induction of apoptosis, and inhibition of topoisomerase [113]. The use of amino naphthoquinones (Rau 008, Rau 010, Rau 015, and Rau 018) with curcumin has been shown to exert synergistic anti-proliferative effects on breast cancer, endometrial, and osteosarcoma cell lines [113].

Doxorubicin as an anti-tumor chemotherapeutic drug combined with curcumin and encapsulated with lipid nanoparticles and polymeric nanoparticles, respectively, has been used to enhance the solubility and overcome rapid clearance of drugs by the reticuloendothelial system. Lipid-coated polymeric nanoparticles (LPNs) provide proper circulating lifetime and delivery of their payload to tumors. Combination therapy with doxorubicin/curcumin nanoparticles has been found to exhibit synergistic cytotoxic effects and fewer side effects. Thus, this combination therapy may be a potential therapeutic strategy for the treatment of osteosarcoma [71].

Codelivery of *ß*-cyclodextrin curcumin and doxorubicin by using a thermosensitive hydrogel, poly(D,L-lactide-co-glycolide)-poly(ethylene-glycol)-poly(D,L-lactide-co-glycolide) showed remarkable improvement in cytotoxicity efficiency and proapoptotic effect of doxorubicin in comparison to using doxorubicin as a single-drug treatment. In addition, the solubility and stability of *ß*-cyclodextrin curcumin were significantly enhanced compared to natural curcumin. The combination of *ß*-cyclodextrin curcumin and doxorubicin showed the greatest Bcl-2 downregulation. While curcumin did not affect expression levels of NF-кB, the protein levels of phosphorylated-IкB, which is an indicator of NF-кB activation [114], were reduced. The expression levels of caspase3, which is an essential molecule in the mitochondrial apoptotic pathway [39], in the groups that were treated with combination therapy were higher compared to the control groups. Furthermore, groups that were treated with combination therapy demonstrated the highest cleaved PARP, an indicator of apoptosis in cancer cells [114, 115].

Dhatchayani et al. demonstrated that the anti-cancer activity of hydroxyapatite nanoparticles with selenite substitution against human osteosarcoma cancer cells was enhanced from 63% to 71% when used in combination with curcumin [116]. Jiang et al. showed that treatment of osteosarcoma MG-63 cells by C086 [4-(4-hydroxy-3-methoxy-phenyl-methyl)], a new structural analog of curcumin, in combination with cisplatin remarkably prevented their proliferation, migration, and invasion while enhancing apoptosis. Additionally, C086+cisplatin inhibited the expression of BMIL1, which is a member of the polycomb group family of transcriptional regulators and exerts an oncogenic role in the development and progression of the tumor. Therefore, combination therapy with C086 and cisplatin has the potential for the treatment of osteosarcoma [117].

## 7. Conclusions

Osteosarcoma remains challenging to treat, and there has been a notable lack of progress in patient survival rates for this aggressive bone cancer. Many chemotherapeutic agents are used to treat osteosarcoma. However, the use of these drugs promotes toxicity and side effects in patients, as well as frequently causing chemoresistance. Curcumin is a natural, polyphenolic nontoxic agent with highly promising anti-oxidant, anti-inflammatory, and anti-cancer properties and fewer side effects, according to available evidence. By targeting several molecular signaling pathways, curcumin exerts its therapeutic effects (Table 1). Although curcumin's in vitro anti-cancer activity has been studied for decades, low bioavailability, poor solubility in aqueous media, and rapid clearance from the plasma have limited its utility in patients. Even high doses of curcumin (8 g/day) administered orally result in substantially low blood levels. Therefore, various delivery systems, such as nanoliposomes, have been developed to enhance curcumin bioavailability and its targeted delivery to cancer cells [118, 119].

In conclusion, curcumin-based treatments may have important applications as novel strategies in the near future to control various diseases, particularly osteosarcoma. Further investigation is needed to define the optimal strategies for curcumin delivery and treatment in human cancers.

## Figures and Tables

**Table 1:** The effect of curcumin on different cellular signaling pathways.

Signaling pathways	Target	Effect	Ref.
RANK/RANKL	ROS producing enzymes, NADPH oxidase, and superoxide dismutase	Suppresses osteoclast formation in response to IL-1*α* by inhibiting the enzymes that generate ROS including NADPH oxidase superoxide dismutase.	[29]
	TNF, IL-1, and IL-8 proinflammatory cytokines	Decreases the proportion of M1‐type macrophages, increases the polarization of macrophages from the M1‐type to the M2‐type phenotype, and has a protective effect on RANKL‐mediated osteoclastogenesis	
	NF-KB (IKK, P65) PKB/AKTC-FOSNFATC1	Attenuates the upregulation of Akt, p65 phosphorylation(ikk), activation of NF-KB, NFATc1, and C-FOS and has a protective effect on RANKL‐mediated osteoclastogenesis	[30]

Notch	Notch-1 Hes-1, Hey-1, and Hey-2	Downregulates the Notch-1 and downstream genes, Hes-1, Hey-1, and Hey-2	[39]

Wnt/β-catenin	β-catenin/Tcf MMP-9*∗* cyclin D1, c-Myc, and survivin	Suppresses the translocation of nuclear *ß*-catenin; inhibits *ß*-catenin/Tcf complex formation; downregulates Wnt target genes (MMP-9, cyclin D1, c-Myc, and surviving); and suppresses the MMP-9 protein, activities Wnt/*β*-catenin leading to the suppression of osteosarcoma cell invasion and metastasis	[46]

Apoptosis	ITPR1	Increases mRNA levels of ITPR1 and induces cytochrome-c-mediated calcium-dependent apoptosis pathway	[50]
	Caspase-3 and Bcl2	Induces the cleavage of caspase-3 and poly adenosine diphosphate-ribose polymerase, decreases the Bcl-2 cellular levels, and increases BAX expression resulting in apoptotic cell death	[52, 53]
	14-3-3*ε* protein	Decreases the 14-3-3*ε* in the nuclear matrix of apoptotic cells and its colocalization with p53, c-FOS, bax, and Bcl-2 in the cytoplasm of osteosarcoma	[52, 55, 58, 59]

Autophagy	JNK	Increases JNK phosphorylation and Beclin1 cellular level	[67]
	Bcl-2	Decreases the cellular level of Bcl-2	

JAK/STAT	JAK2 and STAT3	Inhibites JAK2 and STAT3 phosphorylation	[63]

HIF-1	JNK and ERK	Activates JNK and ERK, induces autophagy, and reverses EMT	[68]

BCL2, B-cell lymphoma 2; EMT, epithelial to mesenchymal transition; ERK, extracellular signal-regulated kinases; ITPR1, inositol 1,4,5-trisphosphate receptor type 1; JNK, c-Jun N-terminal kinases; MMP9, matrix metallopeptidase 9; NADPH, nicotinamide adenine dinucleotide phosphate; NFATC1, nuclear factor of activated *T* cells 1; NF-Κb, nuclear factor-Κb; RANKL, RANKLs ligand; RANK, receptor activator of nuclear factor kappa B; ROS, reactive oxygen species; and TCF, T-cell factor/lymphoid enhancer factor.

## Data Availability

There are no raw data associated with this review.
